# Adhesin Antibody-Grafted Mesoporous Silica Nanoparticles Suppress Immune Escape for Treatment of Fungal Systemic Infection

**DOI:** 10.3390/molecules29194547

**Published:** 2024-09-25

**Authors:** Mengjuan Cheng, Suke Liu, Mengsen Zhu, Mingchun Li, Qilin Yu

**Affiliations:** 1 National Key Laboratory of Intelligent Tracking and Forecasting for Infectious Diseases, College of Life Sciences, Nankai University, Tianjin 300071, China; chengmengjuan2022@163.com (M.C.);; 2 Key Laboratory of Molecular Microbiology and Technology, Ministry of Education, Department of Microbiology, College of Life Sciences, Nankai University, Tianjin 300071, China

**Keywords:** mesoporous silica nanoparticle, adhesin, antibody, *Candida albicans*, systemic fungal infection

## Abstract

Life-threatening systemic fungal infections caused by *Candida albicans* are significant contributors to clinical mortality, particularly among cancer patients and immunosuppressed individuals. The evasion of the immune response facilitated by fungal surface components enables fungal pathogens to evade macrophage attacks and to establish successful infections. This study developed a mesoporous silica nanoplatform, i.e., MSNP-EAP1Ab, which is composed of mesoporous silica nanoparticles grafted with the antibody of *C. albicans* surface adhesin Eap1. The activity of MSNP-EAP1Ab against *C. albicans* immune escape and infection was then evaluated by using the cell interaction model and the mouse systemic infection model. During interaction between *C. albicans* cells and macrophages, MSNP-EAP1Ab significantly inhibited fungal immune escape, leading to the enhanced phagocytosis of fungal cells by macrophages, with phagocytosis rates increasing from less than 8% to 14%. Furthermore, after treatment of the *C. albicans*-infected mice, MSNP-EAP1Ab drastically prolonged the mouse survival time and decreased the kidney fungal burden from >30,0000 CFU/g kidney to <100 CFU/g kidney, indicating the rapid recognition and killing of the pathogens by immune cells. Moreover, MSNP-EAP1Ab attenuated kidney tissue inflammation, with remarkable attenuation of renal immune cell accumulation. This study presents an innovative nanoplatform that targets the *C. albicans* adhesin, offering a promising approach for combatting systemic fungal infections.

## 1. Introduction

Approximately 1.5 million individuals succumb to invasive fungal infections annually on a global scale, with the prevalent culprit being various species of Candida. *Candida albicans* (*C. albicans*) stands out as the primary contributor to candidiasis worldwide [1]. Invasive candidiasis encompasses a variety of disorders, may affect any organ, and refers to deep-seated or disseminated infections (systemic candidiasis), which usually affect patients with impaired host defense mechanisms [2]. Candida bloodstream infections (BSIs) represent the most serious manifestation of candidiasis. The murine model of hematogenously disseminated candidiasis is most commonly used to study systemic candidiasis and to evaluate the efficacy of antifungal therapy. Following intravenous infection, *C. albicans* initially infects almost all organs [3,4], however, the fungal burden in the kidneys increases progressively over time [5], indicating that the kidney acts as the major site of fungal replication [6].

During the inflammatory process, macrophages and neutrophils are crucial effector immune cells involved in fungal eradication and the generation of inflammatory mediators [7,8,9], e.g., tumor necrosis factor α (TNF-α), interleukin-1 (IL-1), and interleukin-6 (IL-6) [10]. The inflammatory factors have the potential to stimulate adaptive immune responses, generate CD4^+^ and CD8^+^ T cells, and bolster antifungal immune activity [11,12,13,14]. While the increased activity of inflammatory monocytes and neutrophils results in hyper-inflammation and lethal kidney pathology [15]. Therefore, effective strategies for alleviating systemic fungal infections involve inhibiting the hyperinflammatory response, controlling fungal proliferation, and maintaining the body’s antifungal activity [16,17,18,19,20]. However, pathogenic fungi evolve complicated immune escape mechanisms, such as the production of surface polysaccharides and proteins to deceive immune cells, and the secretion of proteases to degrade immune response-related molecules [21]. The presence of fungal immune escape frequently compromises the efficiency of immune cells against fungal infections. Up to now, there has been no promising strategy to inhibit fungal immune response.

Mesoporous silica nanoparticles (MSNs) have emerged as a versatile imaging platform in recent years, offering customizable size and morphology, diverse surface chemistry, biocompatibility, and other advantageous physicochemical properties [22,23,24]. Simultaneously, extensive research has been conducted on MSN-based gating systems to integrate pore-capping, drug-loading, and targeting abilities (Table S1) [25,26,27,28]. Nevertheless, few MSN delivery platforms are tailored for targeting *C. albicans*, underscoring the critical necessity to develop MSN platforms that are responsive to fungal infections in order to combat these pathogens.

Eap1 is a novel adhesin of *C. albicans*, capable of mediating adhesion to both polystyrene and epithelial cells. Its expression is under the regulation of the transcription factor Efg1p, which is essential for hyphal formation, adhesion to and invasion of multilayered human epidermal tissue, and virulence in a murine model [29,30]. Importantly, in both hyphal and yeast cells, Eap1 could be highly expressed and exposed on the surface of *C. albicans* cells, endowing it with an ideal surface antigen for the specific targeting of this pathogen. However, up to now, there has been no study focusing on it as a target of antifungal strategies.

**Figure 1:**
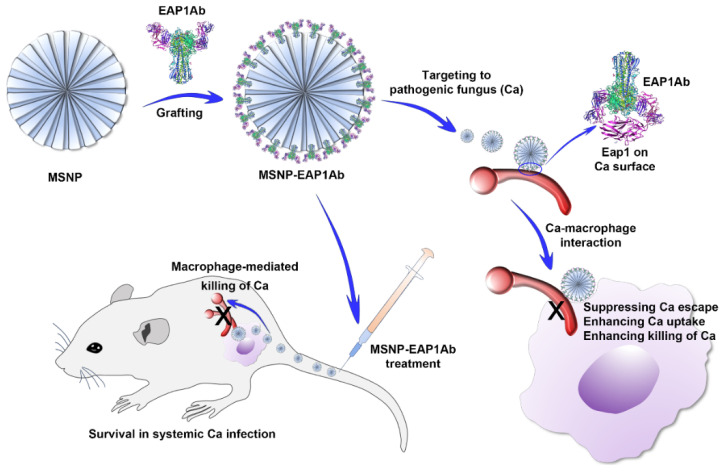
A scheme illustrating the preparation of MSNP-EAP1Ab, suppression of *C. albicans* (Ca) immune escape, and enhancement of macrophage-mediated Ca killing against systemic fungal infection.

In this study, to avoid the immune escape of the life-threatening *C. albicans*, we developed a mesoporous silica nanoparticle platform (MSNP) grafted by the adhesin antibody for treatment of fungus-induced systemic infections (Figure 1). The MSNP was firstly prepared by the surfactant-mediated formation of silica pores, and then grafted by the *C. albicans* adhesin Eap1 antibody EAP1Ab to obtain MSNP-EAP1Ab. During the interaction between the pathogenic fungus *C. albicans* (Ca) and the macrophages, MSNP-EAP1Ab strongly suppressed the immune escape of Ca, leading to the enhanced recognition of Ca and consequent efficient killing of Ca by macrophages. During the systemic infection by Ca, the MSNP-EAP1Ab treatment efficiently causes the macrophage-mediated killing of Ca, leading to the prolonged survival of mice with the life-threatening Ca infection. This study provided a fungal antibody-grafted nanoplatform for avoiding fungal immune escape to treat dangerous fungal systemic infections.

## 2. Results

### 2.1. Characterization of MSNP and MSNP-EAP1Ab

**Figure 2:**
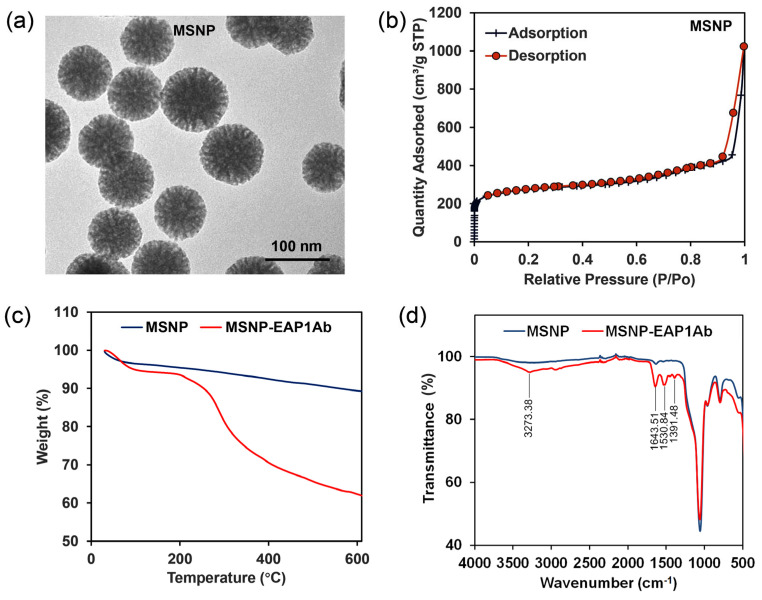
Characterization of the prepared MSNP and MSNP-EAP1Ab. (**a**) The TEM image of MSNP. (**b**) The adsorption–desorption curve of MSNP. (**c**) TGA curves of MSNP and MSNP-EAP1Ab. (**d**) FT-IR spectra of MSNP and MSNP-EAP1Ab.

As revealed by the TEM image, the initial MSNP had regular round-like morphology, with the radically distributed pores in the nanoparticles (Figure 2a). The adsorption–desorption curve revealed a sudden change of adsorbed quantity when the relative pressure increased to 0.95 (Figure 2b), with the average pore diameter at 6.239 nm. TGA analysis showed that the weight of the MSNP nanoparticles decreased to 90%, while that of MSNP-EAP1Ab decreased to 62% when the temperature reached 600 °C (Figure 2c). This indicated that approximately 28% of the antibody was grafted onto the MSNP. In addition, as shown in the FT-IR spectra, the MSNP-EAP1Ab nanoparticles had differential absorption peaks of 3723.38 cm^−1^, 1643.51 cm^−1^, 1530.84 cm^−1^, and 1391.48 cm^−1^ (Figure 2d), confirming the presence of -CO-NH- in MSNP-EAP1Ab. This indicated that EAP1Ab was successfully grafted onto the MSNP.

### 2.2. MSNP-EAP1Ab Enhances Both Phagocytosis and Cytokine Secretion in RAW264.7 Cells

**Figure 3:**
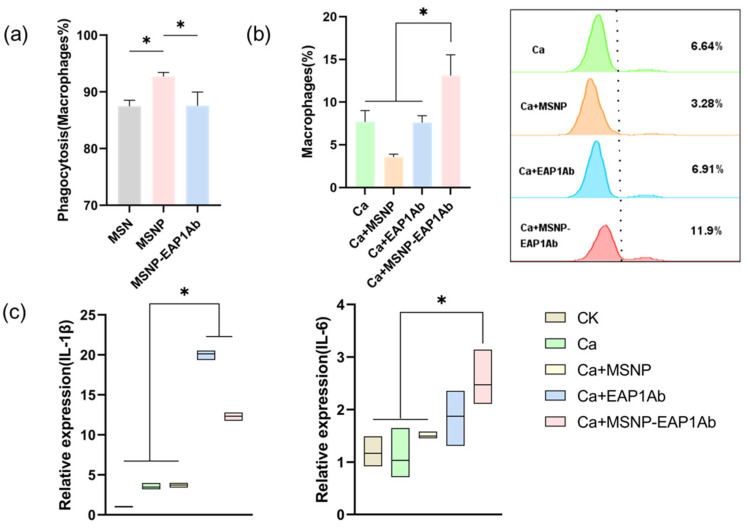
MSNP-EAP1Ab enhances both phagocytosis and cytokine secretion in RAW264.7 cells. (**a**) Endocytosis of MSN, MSNP, and MSNP-EAP1Ab was observed (6 h). (**b**) The impact of MSNP and MSNP-EAP1Ab on macrophage phagocytosis post-*C. albicans* infection. (**c**) The impact of MSNP and MSNP-EAP1Ab on the transcription levels of IL-1β and IL-6 following *C. albicans* infection. The asterisk indicates a significant difference between the two groups (*p* < 0.05).

Macrophages are vital players in the host’s innate immune response. Phagocytosis, defined as the uptake of particles greater than 0.5 μm, is a significant process in the innate immune response [31]. After co-culturing with FITC-labeled MSN, MSNP, MSNP-EAP1Ab, and RAW264.7 cells, the macrophages’ uptake of nanomaterials was assessed via flow cytometry (Figure 3a). The endocytosis rate of MSN exceeded 85%, and, following P modification, MSNP-EAP1Ab showed over 90% endocytosis. However, the endocytosis rate of MSNP-EAP1Ab in RAW264.7 cells decreased to 85%–90%. Subsequent experiments will be conducted using MSNP and MSNP-EAP1Ab. Based on the aforementioned co-culture of nanomaterials with RAW264.7 cells, we infected them with GFP-labeled *C. albicans* to assess the impact of nanomaterials on macrophage phagocytosis using flow cytometry. As shown in Figure 3b, it is evident that MSNP significantly decreased macrophage phagocytosis, whereas MSNP-EAP1Ab notably increased it.

Macrophages play a critical role in inflammation by producing cytokines like IL-1β and IL-6 to facilitate fungal killing. Following the infection of macrophages and nanomaterial co-cultures by *C. albicans*, we evaluated IL-1β, IL-6 and TNF-α transcription levels using RT-qPCR (Figure 3c and Figure S1). Our findings indicated that MSNP-EAP1Ab demonstrated the ability to enhance the expresssion of these cytokines, promoting *C. albicans* elimination. Conversely, upon *C. albicans* infection, EAP1Ab resulted in excessive IL-1β transcription, triggering heightened cellular inflammation and increasing the likelihood of pyroptosis.

### 2.3. MSNP-EAP1Ab Suppresses Mouse Death and Kidney Damage Induced by Systemic C. albicans Infection

**Figure 4:**
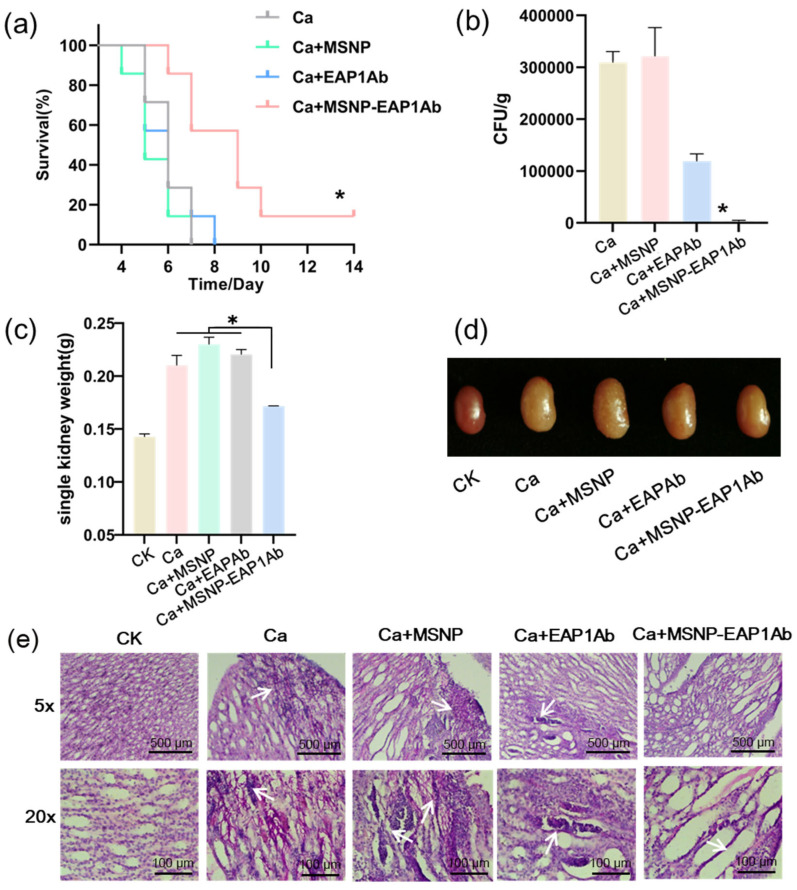
MSNP-EAP1Ab mitigates kidney damage induced by systemic *C. albicans* infection. (**a**) Survival curves of the mice (n = 10 mice/group). The mice were infected by the fungal cells (5 × 10^6^ cells/mouse) via tail intravenous injection on day 0, mice were divided into groups and treated with MSNP, EAP1Ab, or MSNP-EAP1Ab on day 1, the survival of mice was monitored for 14 days. (**b**) Fungal burdens at day 5 post-infection were presented per kidney or gram basis (n = 6 kidneys/group). (**c**) Weight of the kidneys at day 5 post-infection (n = 6 kidneys/group). (**d**) The morphology of kidneys at day 5 post-infection. (**e**) Histopathologic findings: PAS staining. The asterisk indicates a significant difference between the two groups (*p* < 0.05).

In a mouse model of systemic infection, the kidney emerges as the primary site of infection, underscoring the significance of mitigating inflammation within the kidney for the effective treatment of *C. albicans* systemic infection [32,33]. Twenty-four hours following systemic infection of mice by *C. albicans*, administering MSNP-EAP1Ab via a tail vein injection significantly prolonged the survival time of the mice (Figure 4a). On the fifth day post-infection, the mice underwent kidney weight measurements. The data presented in Figure 4b revealed that the group treated with MSNP-EAP1Ab exhibited a more vibrant kidney color and reduced swelling, whereas the group treated with MSNP alone displayed pronounced kidney swelling and renal nodules. Simultaneously, the kidneys were trisected and revealed a reduced fungal burden in the kidneys of mice treated with MSNP-EAP1Ab (Figure 4c). Analysis of kidney sections through Periodic Acid-Schiff staining revealed that mice treated with MSNP exhibited a considerable presence of hyphae at the renal pelvis site, leading to the disruption of their tissue morphology. In contrast, mice treated with EAP1Ab showed a substantial accumulation of immune cells, causing severe local kidney damage, but without notable hyphal growth. Mice receiving the MSNP-EAP1Ab treatment maintained relatively intact tissue morphology, with an enhanced and evenly distributed population of immune cells (Figure 4d).

### 2.4. MSNP-EAP1Ab Enhances the Cytotoxicity of Renal Immune Cells without Inducing Excessive Inflammation

**Figure 5:**
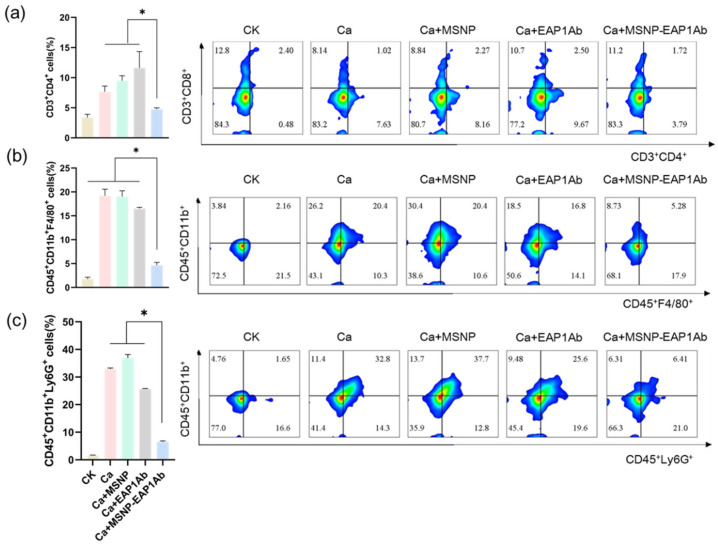
MSNP-EAP1Ab attenuates the accumulation of renal immune cells in Ca-infected mice. (**a**) The CD3+CD4+ T cell levels in the kidneys at day 5 post-infection (n = 4/group). (**b**) The CD45+CD11b+F4/80+ macrophage levels in the kidneys at day 5 post-infection (n = 4/group). (**c**) The CD45+CD11b+Ly6G+ neutrophils levels in the kidneys at day 5 post-infection (n = 4/group). The asterisk indicates a significant difference between the two groups (*p* < 0.05).

Insufficient early inflammatory cell-mediated response impairs the ability of mice to control fungal growth. Conversely, an excessive fungal growth elicits a heightened inflammatory response in mice, ultimately culminating in multi-organ failure [34]. In this study, flow cytometry was employed to evaluate the levels of CD3^+^CD4^+^ T cells (Figure 5a), CD45^+^CD11b^+^F4/80^+^ macrophages (Figure 5b), and CD45^+^CD11b^+^Ly6G^+^ neutrophils (Figure 5c) in the kidney. Excessive proliferation of these immune cells can result in an intensified inflammatory response, thereby causing damage and renal failure. MSNP-EAP1Ab effectively suppressed the proliferation of inflammatory cells and demonstrated a notable fungicidal impact when compared to the control group.

**Figure 6:**
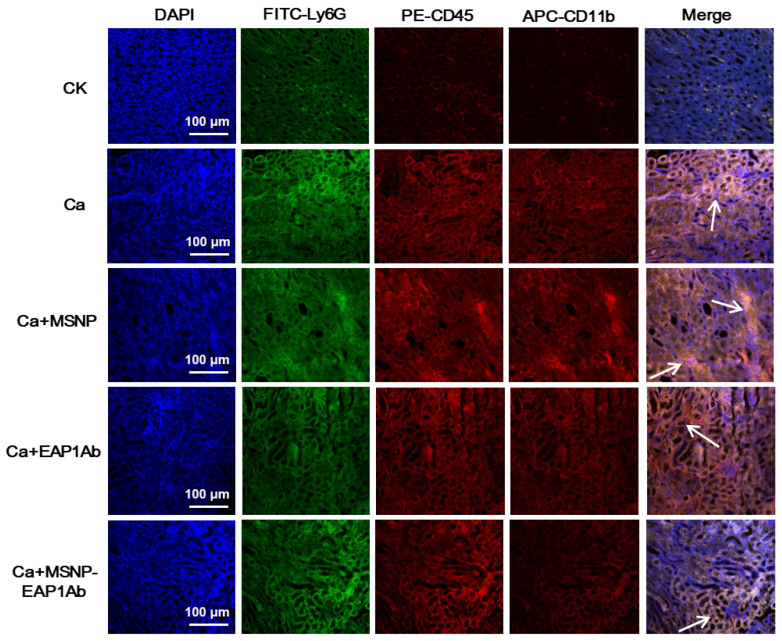
MSNP-EAP1Ab attenuates the in situ localization of pro-inflammatory immune cells in Ca-infected kidneys. The white arrows indicated the representative sites displaying both FITC-Ly6G, PE-CD45 and APC-CD11b.

Immunofluorescent staining of neutrophils from the kidneys was then performed to further detect the distribution of immune cells. As revealed by observation using confocal microscopy, while the groups of Ca, Ca+MSNP, and Ca+EAP1Ab exhibited dense distribution of the pro-inflammatory immune cells (Ly6G^+^, CD45^+^ and CD11b^+^) in the kidney tissues, the MSNP-EAP1Ab only had gentle distribution of these immune cells (Figure 6). These results are consistent with the findings obtained through flow cytometry analysis (Figure 5). Hence, the treatment of MSNP-EAP1Ab severely attenuated the accumulation of pro-inflammatory immune cells, leading to the alleviation of inflammation in the infected kidneys.

## 3. Discussion

The human fungal pathogen *C. albicans* causes invasive candidiasis, a condition marked by fatal organ failure resulting from widespread fungal proliferation and inflammatory harm. Among peripheral organs, the kidneys offer the most conducive environment for fungal growth and the transition to a hyphal form [35,36]. An efficient inflammatory response is crucial for combating infections, however, the usual reaction to *C*. *albicans* results in tissue damage, intensifying the pathological consequences of the infection. Therefore, there is a requirement to evolve sophisticated and improved treatment strategies.

Research on intelligent drug delivery systems in the biomedical sector is anticipated to improve drug effectiveness and mitigate side effects at the sites of diseases. The distinctive characteristics of mesoporous silica nanoparticles (MSNs), including their capacity for stable covalent attachment to recognition groups like antibodies or aptamers, offer a myriad of possibilities for developing drug delivery platforms and biosensors. Currently, numerous drug delivery systems are available, such as PEG-MSN-Stattic, Chl-MSN, and MSN-NH_2_/RC [37,38,39,40]. Targeted delivery involves the following mechanism: the carrier containing the drug enters the bloodstream, circulates through the body, and accumulates exclusively in the area of the lesion [41]. Consequently, the nanoplatform incorporating EAP1Ab demonstrates superior delivery efficiency and targeting capabilities.

## 4. Materials and Methods

### 4.1. Materials

The Periodic Acid-Schiff Staining Kits (C0142M) were purchased from Beyotime, Shanghai, China. The primers for RT-qPCR were synthesized by Sangon Biotechnology (Sangon Biotech Co., Ltd., Shanghai, China). The 4′, 6 diamidino-2-phenylindole (DAPI, C1002) was purchased from Beyotime, China. The PE Anti-Mouse CD45 Antibody (E-AB-F1136D) was purchased from Elabscience, Wuhan, China. The FITC anti-mouse F4/80 (123107), APC anti-mouse IFN-γ (505809), FITC anti-mouse Ly-6G (127605), APC anti-mouse/human CD11b (101211), and APC anti-mouse TNF-α (506307) were purchased from BioLegend (Biotechnology Co., Ltd., San Diego, CA, USA). The wild-type *C. albicans* strain SC5314 was purchased from ATCC, Manassas, VA, USA. The GFP-labeled *C. albicans* strain CaGFP was constructed by transformation of the wild-type *C. albicans* strain BWP17 with the Erg6-localizing plasmid pErg6-GFP. The RAW264.7 macrophages were purchased from the Cell Resource Center, Beijing, China. The antibody against the *C. albicans* adhesin Eap1 (i.e., EAP1Ab) was prepared by immunization of the rabbits with the purified the Eap1 antigen, followed by serum collection and antibody purification.

### 4.2. Preparation and Characterization of the Nanoparticles

The initial MSNP was prepared by using tetraethyl orthosilicate (TEOS) as the silica source, and hexadecyltrimethylammonium bromide (CTAB) as the template agent. Briefly, 400 mg of CTAB was dissolved in 100 mL of distilled water. Under constantly magnetic stirring, 448 μL of the 100 mg/mL NH_4_Cl solution, 100 μL of the 2 mol/L NaOH solution, and 1.828 mL of mesitylene were then added into the CTAB solution. The mixture was stirred at 8000 rpm and 78 °C for 30 min, followed by the addition of 1.5 mL TEOS and further stirring for another 1 h. A total of 100 μL of (3-aminopropyl)triethoxysilane (APTES) were then added into the reaction solution, and the mixture was further stirred for 30 min. The nanoparticles were centrifuged at 12,000 rpm for 10 min, and washed by ethanol and distilled water, obtaining the initial MSNP.

To graft the MSNP by EAP1Ab, 50 mg of the MSNP were suspended in 50 mL of distilled water, and then 100 μL of glutaric dialdehyde were added into the suspension. The mixture was magnetically stirred at 750 rpm for 6 h. The activated nanoparticles were harvested by centrifugation at 12,000 rpm for 10 min, and washed by distilled water three times. The nanoparticles were further suspended in 50 mL of PBS (20 mM, pH = 7.2) containing 50 mg EAP1Ab. The mixture was also stirred at 800 rpm for 12 h under 4 °C for 12 h, followed by centrifugation with the same condition. The nanoparticles were washed three times in distilled water and dried by a freeze vacuum dried, obtaining MSNP-EAP1Ab.

The morphology and pore sizes of MSNP were characterized by using a transmission electron microscope (TEM, Tecnai T12, FEI, Hillsboro, OR, USA) and a Brunauer-Emmett-Teller (BET) pore size analyzer (ASAP 2460, Micromeritics, Norcross, GA, USA), respectively. The thermogravimetric analysis (TGA) of MSNP and MSNP-EAP1Ab was performed using a TG/DTA Instrument (STA 8000, PerkinElmer, Waltham, MA, USA) under the nitrogen flow. The Fourier Transform Infrared Spectoscopy (FT-IR) spectra of the nanoparticles were performed by using the FT-IR analyzer (Nicolet iS20, Thermo Fisher Scientific, Waltham, MA, USA).

### 4.3. Animals

All animal experiments were approved by the Animal Care and Use Committee of Nankai University. Fifty 5-week-old ICR pathogen-free (SPF) female mice, weighing between 18 g and 20 g, were chosen and randomly divided into groups of 10 replicates. The mice were maintained in a 12-h light-dark cycle with ad libitum access to food and water.

### 4.4. Cell Culture

Dulbecco’s Modified Eagle’s Medium (DMEM) with high glucose (HyClone, Logan, UT, USA), enriched with 10% heat-inactivated fetal bovine serum (FBS) (HyClone, Logan, UT, USA) was used for the growth of RAW264.7 cells in an atmosphere containing 5% CO_2_ at 37 °C without any penicillin or streptomycin, and 5 × 10^6^ cells were inoculated into a fresh 10-cm cell-culture dish (Corning-Costar, Corning, NY, USA). Upon 80% confluence, RAW 264.7 cells were sub-cultured serially to be used for the following experiments.

### 4.5. Cellular Phagocytosis

The day before the experiment, RAW264.7 cells were seeded onto a 24-well plate. Once the cells adhered to the surface, MSNP and MSNP-EAP1Ab were sequentially added. After a 6-h incubation period, the supernatant was aspirated, and the cells were washed twice with PBS. Subsequently, *Erg6*^−/−^-GFP *C. albicans* (MOI 5) was added, followed by a 40-min co-incubation at 37 °C with 5% CO_2_. The cells were then harvested, and the phagocytic activity was assessed by measuring the fluorescence signal of GFP using flow cytometry after appropriate steps of washing, centrifugation, and resuspension.

### 4.6. MSN Endocytosis Efficiency

The RAW264.7 cells were plated onto a 24-well plate and incubated with FITC-labeled MSNP and MSNP-EAP1Ab overnight on a shaker. Subsequently, the supernatant was discarded, and the cells were resuspended after centrifugation at 12,000 rpm for 2 min. The cells were then grouped and added back onto a 24-well plate for a 6-h incubation period. Following this, the cells were collected by centrifugation at 1200 rpm, washed, and resuspended in PBS. The fluorescence signal intensity of macrophages was later detected using flow cytometry.

### 4.7. Reverse Transcriptase PCR

The macrophages (2.0 × 10^5^ cells/well) were seeded overnight in a six-well plate. Total RNA was extracted from treated cells according to the manufacturer’s instructions (TransGen Biotech, Beijing, China) and used for cDNA synthesis (One-Step gDNA Removal, TransGen Biotech). PCR was then performed using primer pairs specific for TNFα, IL-1β, IL-6, and β-actin (the primer sequences are shown in Table 1). The amplified products were electrophoresed using 1.5% agarose gel and stained with ethidium bromide before visualization under a UV lamp.

### 4.8. Systemic Infection

To evaluate the therapeutic effect of MSNP-EAP1Ab, the mice (except for the control group) were infected by the fresh *C. albicans* SC5314 cells with intravenous inoculation at the tail vein on day 0. After 24 h, the mice were injected with MSNP, EAP1Ab, and MSNP-EAP1Ab via the tail vein based on their respective group assignments. The survival of the infected mice was recorded from day 0 to day 14. On day 4, the kidneys of three mice in each group were sampled and weighed. The kidneys were further broken by Dounce homogenizers, and the numbers of the fungal cells in the obtained homogenates were determined using colony-forming unit assays on plates of yeast extract-peptone-dextrose medium.

### 4.9. Flow Cytometry Assays

On the fifth day of systemic infection, the kidneys of mice were harvested. The kidney tissues were digested into a cell suspension, which was subsequently washed twice with phosphate-buffered saline (PBS), and then resuspended. The cells were diluted for staining with a fluorescent dye at a ratio of 1:1000, incubated in the dark at 4 °C for 30 min, followed by resuspension after two washes with PBS for detection.

### 4.10. Paraffin Immunofluorescence

On the fifth day of systemic infection, the mouse kidneys were harvested, fixed in 4% paraformaldehyde for 24 h, and subsequently processed through dehydration, transparency, and embedding in paraffin blocks. The resulting sections were cut into slices of 5 to 10 microns thickness, followed by deparaffinization and rehydration using xylene and ethanol gradients after a 2-h baking period. A citrate antigen retrieval solution (pH 6.0) was prepared, and antigen retrieval was conducted in a microwave oven. Following natural cooling, the tissues were washed three times with PBS buffer for 5 min each. A dye, diluted at a ratio of 1:500, was then meticulously added to the tissue and left to stain in darkness for 30 min before observation under a confocal microscope.

### 4.11. Statistical Analysis

Most of the experiments were performed with three replicates (n = 3), except the experiment of systemic infection (n = 10). The data were shown with the means ± the standard errors. The differences between the groups were analyzed by the ANOVA test and the Student’s *t*-test (*p* < 0.05) via SPSS 22 software (IBM, Armonk, NY, USA).

## 5. Conclusions

In conclusion, this study developed a novel adhesin antibody-grafted nanoplatform for treatment of the body from life-threatening systemic fungal infections. MSNP was synthesized through the surfactant-mediated creation of silica pores, followed by the grafting of Msnp-EAP1Ab with the Eap1 antibody specific to the *C. albicans* adhesin EAP1Ab. Upon interaction with macrophages, MSNP-EAP1Ab effectively suppressed the immune escape of *C. albicans*, leading to the increased recognition and killing of the pathogen. In the systemic *C. albicans* infection model, treatment with MSNP-EAP1Ab successfully triggered the macrophage-mediated killing of *C. albicans*, resulting in the prolonged survival time of mice facing life-threatening fungal infections and attenuation of kidney inflammation. In conclusion, this study introduced a novel nanoplatform utilizing a fungal antibody grafted nanoplatform to counteract fungal immune evasion against severe systemic fungal infections.

## Figures and Tables

**Table 1:** Nucleotide primer sequences used for PCR amplification.

Primer	Sequence	
	Sense	Antisense
Mouse-TNFα	GGTGCCTATGTCTCAGCCTCTT	GCCATAGAACTGATGAGAGGGAG
Mouse-IL-1β	TGGACCTTCCAGGATGAGGACA	GTTCATCTCGGAGCCTGTAGTG
Mouse-IL-6	TACCACTTCACAAGTCGGAGGC	CTGCAAGTGCATCATCGTTGTTC
Mouse-β-actin	CATTGCTGACAGGATGCAGAAGG	TGCTGGAAGGTGGACAGTGAGG

## Data Availability

The original contributions presented in the study are included in the article/Supplementary Material.

## Supplementary Materials

The following supporting information can be downloaded at: https://www.mdpi.com/article/10.3390/molecules29194547/s1, Figure S1. The impact of MSNP, and MSNP-EAP1Ab on the transcription levels of TNFα following *C. albicans* infection; Table S1. The diverse applications of MSNs as drug delivery carriers across various fields.
