# Supplementary material for: Adhesin Antibody-Grafted Mesoporous Silica Nanoparticles Suppress Immune Escape for Treatment of Fungal Systemic Infection
Source: Molecules. 2024 Sep 25;29(19):4547. doi: 10.3390/molecules29194547 (PMC11478059; doi:10.3390/molecules29194547)
Supplement: Supplementary file 1 [file molecules-29-04547-s001.zip › molecules-3189522-supplementary.pdf]

Adhesin Antibody-Grafted Mesoporous Silica Nanoparticles Suppress Immune Escape for Treatment of Fungal Systemic Infection

Mengjuan Cheng [1,2], Suke Liu [1], Mengsen Zhu [1], Mingchun Li [1] and Qilin Yu [1,2,*]

[1] National Key Laboratory of Intelligent Tracking and Forecasting for Infectious Diseases, College of Life Sciences, Nankai University, Tianjin 300071, China; chengmengjuan2022@163.com (M.C.)
[2] Key Laboratory of Molecular Microbiology and Technology, Ministry of Education, Department of Microbiology, College of Life Sciences, Nankai University, Tianjin 300071, China
* Correspondence: yuqilin@mail.nankai.edu.cn

Table S1 The diverse applications of MSNs as drug delivery carriers across various fields

Category	Medicine	Platform	Outcome	Reference
Breast Cancer	Paclitaxel (PTX)	MSNs-NH2-FA-RGD	The inhibitory efficacy of PTX@MSNs-NH2-FA-RGD on MCF-7 was 1.6 times than that of free PTX.	Doi: 10.3389/fphar.2020.00898
Gram-Positive Bacteria	Vancomycin	MSNs	MSNs ⊂ Van showed unambiguous antibacterial efficacy without decrease in cell viability of macrophage-like cells.	Doi: 10.1021/am403940d
Parkinson's disease	Pramipexole	MCM-41	Pramipexole loading to surface-coated MCM-41 exhibited efficiency in reducing H2O2induced toxicity in neuronal SH-SY5Y cells as well as oxidative damage in PD	Doi: 10.1016/j.jddst.2019.02.008
Rheumatoid Arthritis	dexamethasone (Dex)	RMSNs	Dex-loaded RMSNs sustained significant anti-inflammatory effects and recovery of cartilage over a period of 8 weeks.	Doi: 10.3390/pharmaceutics14050985
Systemic fungal infection	EAP1Ab	MSNP	MSNP-EAP1Ab attenuated kidney tissue inflammation, with remarkable attenuation of renal immune cell accumulation.	Reference this article.

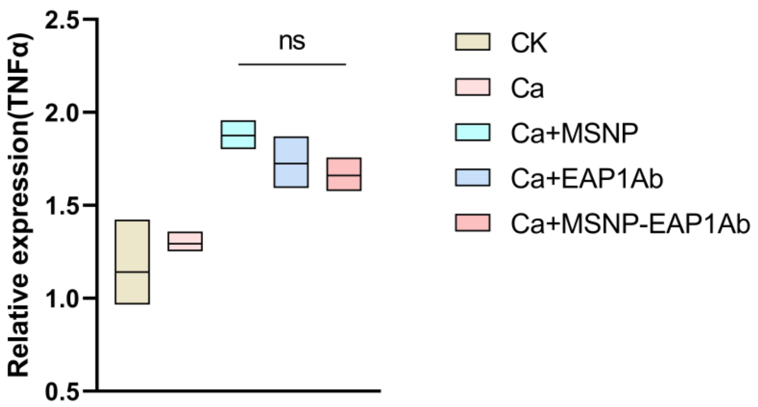

Figure S1. The impact of MSNP, and MSNP-EAP1Ab on the transcription levels of TNF α following C. *albicans* infection.
